# Potential vector switching in the evolution of *Bursaphelenchus xylophilus* group nematodes (Nematoda: Aphelenchoididae)

**DOI:** 10.1002/ece3.7033

**Published:** 2020-12-01

**Authors:** Noritoshi Maehara, Natsumi Kanzaki, Takuya Aikawa, Katsunori Nakamura

**Affiliations:** ^1^ Department of Forest Entomology Forestry and Forest Products Research Institute Tsukuba Japan; ^2^ Kansai Research Center Forestry and Forest Products Research Institute Kyoto Japan; ^3^ Tohoku Research Center Forestry and Forest Products Research Institute Morioka Japan

**Keywords:** *Acalolepta fraudatrix*, broad‐leaved tree, *Bursaphelenchus doui*, conifer, phoretic stage

## Abstract

To show the importance of vector switching of nematodes in the evolution of the *Bursaphelenchus xylophilus* group, we tested a hypothesis that “*Bursaphelenchus doui* (or its ancestor) was transferred by *Acalolepta fraudatrix*, *Acalolepta sejuncta*, and/or *Monochamus subfasciatus* (or their ancestral species) from broad‐leaved trees to conifers, switched vectors from these cerambycid beetles to *Monochamus* beetles in conifers, and then evolved into the common ancestor of *Bursaphelenchus mucronatus* and *B. xylophilus*.” We used a simple nematode‐loading method to beetles and produced 20 binary combinations of five *B. xylophilus* group species and four cerambycid beetle species in the tribe Lamiini. The affinity of the nematodes for the beetles was examined based on phoretic stage formation of the nematodes. Phoretic stages of *B. doui* appeared in all beetle species examined, namely *Acalolepta luxuriosa*, *Psacothea hilaris*, *A. fraudatrix*, and *Monochamus alternatus*, although the affinity of the nematode for *M. alternatus* was weak. This finding indicates that *B. doui* could switch vectors to conifer‐using *Monochamus* beetles after transfer by *A. fraudatrix* from broad‐leaved trees to conifers. We conclude that vector switching of nematodes could have potentially happened during the evolutionary history of the *B. xylophilus* group.

## Graphical abstract

To clarify the role of vector switching of nematodes in the evolution of the *Bursaphelenchus xylophilus* group, we used a simple nematode‐loading method to beetles and produced 20 binary combinations of five *B. xylophilus* group species and four cerambycid beetle species in the tribe Lamiini. The affinity of the nematodes for the beetles was examined based on phoretic stage formation of the nematodes. The results showed that vector switching of nematodes could have potentially happened during the evolutionary history of the *B. xylophilus* group.

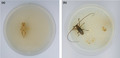

## 1 INTRODUCTION

Many *Bursaphelenchus* nematodes belonging to the *Bursaphelenchus xylophilus* group sensu Braasch et al. (2009) are associated with cerambycid beetles in the tribe Lamiini. The pinewood nematode *B. xylophilus* (Steiner & Buhrer) Nickle, the causal agent of pine wilt disease (Kiyohara & Tokushige, 1971), and its closest relative, *Bursaphelenchus mucronatus* Mamiya and Enda are vectored by *Monochamus* cerambycid beetles (Linit, 1988; Mamiya & Enda, 1972, 1979; Morimoto & Iwasaki, 1972; Penas et al., 2006; Sousa et al., 2001, 2002; Tomminen, 1990). Also, nematode/vector combinations of *B. xylophilus* group species include, *Bursaphelenchus conicaudatus* Kanzaki, Tsuda & Futai/*Psacothea hilaris* (Pascoe) (Kanzaki et al., 2000); *Bursaphelenchus luxuriosae* Kanzaki & Futai and *Bursaphelenchus acaloleptae* Kanzaki, Ekino, Maehara, Aikawa, & Giblin‐Davis/*Acalolepta luxuriosa* (Bates) (Kanzaki et al., 2020; Kanzaki & Futai, 2003a); and *Bursaphelenchus firmae* Kanzaki, Maehara, Aikawa, & Matsumoto/*Monochamus grandis* Waterhouse (Kanzaki et al., 2012). In contrast, *Bursaphelenchus doui* Braasch, Gu, Burgermeister, & Zhang is found in association with several species of cerambycid beetles, that is, *Acalolepta fraudatrix* (Bates) (Kanzaki et al., 2013), *Acalolepta sejuncta* (Bates) (Aikawa et al., 2020), *Monochamus subfasciatus* (Bates) (Kanzaki et al., 2008), and *Monochamus saltuarius* (Gebler) (Aikawa et al., 2020).

The habitats for the above nematodes are determined by their vector beetles. Therefore, *B. conicaudatus* and *B. luxuriosae* are found in broad‐leaved trees, and *B. xylophilus*, *B. mucronatus*, and *B. firmae* inhabit conifers. By contrast, *B. doui* is present in both broad‐leaved trees (Han et al., 2009) and conifers (Kanzaki et al., 2008) because vectors for this species, *A. fraudatrix*, *A. sejuncta*, and *M. subfasciatus*, use both. *Monochamus saltuarius* is also a vector but inhabits only coniferous species.

Kanzaki and Futai (2002a) proposed that the ancestral species of *B. xylophilus* group, which had originated in the Eurasian Continent, obtained the ability to use tree species of family Pinaceae instead of broad‐leaved ones and expanded their distribution throughout the coniferous forests ranging widely in the ancient Eurasia‐North America continent. Molecular phylogenetic analyses inferred from rRNA gene segments D2‐D3 LSU in Figure 3 of Kanzaki et al. (2012) showed that nematodes in conifers evolved from nematodes in broad‐leaved trees. The higher genetic diversity of *B. mucronatus* could be the result of an earlier origin in Eurasia, and *B. xylophilus* could evolved recently from a *B. mucronatus* population in North America through geographical or reproductive isolation (Pereira et al., 2013). For this evolutionary process, cerambycid beetles must have transferred nematodes from broad‐leaved trees to conifers. We hypothesized that “*B. doui*, or its ancestor, was transferred by *A. fraudatrix*, *A. sejuncta*, and/or *M. subfasciatus* (or ancestral species of these beetles) from broad‐leaved trees to conifers, switched vectors from these beetles to *Monochamus* beetles, that is, *M. saltuarius*, in conifers, and later evolved into the common ancestor of *B. mucronatus* and *B. xylophilus*.”

The life cycle of *B. xylophilus* is divided into propagative and dispersal phases. The fourth‐stage dispersal juvenile (dauer juvenile; J_IV_) of *B. xylophilus* is vital in the nematode life cycle as the phoretic stage carried by beetles. *Bursaphelenchus xylophilus* J_IV_ develops when late pupae and callow adults of *Monochamus* beetles are present (Maehara & Futai, 1996, 2001; Morimoto & Iwasaki, 1973; Necibi & Linit, 1998; Ogura & Nakashima, 2002) and enters the tracheae of the beetles. Phoretic stages of *B. mucronatus* (Mamiya & Enda, 1979), *B. conicaudatus* (Kanzaki & Futai, 2001), and *B. firmae* (Kanzaki et al., 2012) are also J_IV_. Phoretic stages of *B. luxuriosae* (Ekino et al., 2017; Kanzaki et al., 2009) and *B. acaloleptae* (Kanzaki et al., 2020) are the phoretic adults (PA) and *B. doui* (Kanzaki et al., 2013) both J_IV_ and PA. J_IV_ of *B. conicaudatus* and PA of *B. luxuriosae* are also induced by their vector beetles (Maehara et al., 2013). Moreover, J_IV_ of *B. xylophilus* is induced not only by its primary vector *M. alternatus* Hope but also by nonvector *P. hilaris*, although the numbers and the percentages of J_IV_ are markedly higher in the former than in the latter (Maehara & Futai, 2001). The third‐stage dispersal juveniles (J_III_) of *B. xylophilus* molt into J_IV_ in response to long‐chain C16 and C18 fatty acid ethyl esters that are secreted from the body surface of *M. alternatus*, specifically during adult eclosion (Zhao et al., 2013, 2014). Thus, J_IV_ and PA are specific and essential to vector association.

In the present study, our objective was to test the above hypothesis and demonstrate the importance of vector switching of nematodes in the evolution of the *B. xylophilus* group. We used a simple nematode‐loading method to cerambycid beetles (Maehara & Kanzaki, 2016), which could be used to examine the affinity of nematodes for not only their vectors but also nonvectors, and produced 20 binary combinations of five *B. xylophilus* group species and four cerambycid beetle species in the tribe Lamiini. These nematode/beetle combinations were examined for the effects of the vector and nonvector beetles on the formation of the nematode phoretic stages, that is, J_IV_ and PA.

## 2 MATERIALS AND METHODS

### 2.1 Beetle cultures

Japanese black pine trees, *Pinus thunbergii* Parl., infested with *M. alternatus* larvae were felled and cut at the Chiyoda Experimental Station of the Forestry and Forest Products Research Institute (FFPRI), Kasumigaura, Ibaraki, Japan in spring 2009. Logs were placed in a screen cage at the FFPRI, Tsukuba, Ibaraki, Japan, and adults of *M. alternatus* that emerged from the logs in May to June 2009 were collected. Adults of *A. luxuriosa* were captured in May to July 2009 from *Aralia elata* (Miquel) Seemann trees in experimental fields at the FFPRI, Tsukuba and at the Tohoku Research Center, FFPRI, Morioka, Iwate, Japan. Adults of *P. hilaris* were collected in May and June 2009 from a fig tree, *Ficus carica* L., planted in an experimental field at the FFPRI, Tsukuba. *Monochamus alternatus*, *A. luxuriosa*, and *P. hilaris* were allowed to oviposit on *P. densiflora* Sieb. and Zucc. logs cut about 1 week prior, fresh hand‐rolled leaves of *A. elata* (Akutsu, 1985), and fresh hand‐rolled leaves of *Morus bombycis* Koidzumi (Maehara et al., 2013), respectively. Eggs of *M. alternatus* were harvested from the logs by a chisel, and those of *A. luxuriosa* and *P. hilaris* were collected by opening the hand‐rolled leaves. These eggs were put on wet filter paper with distilled water at 25°C in the dark until they hatched. Artificial diets were modified from the diet for *M. alternatus* proposed by Kosaka and Ogura (1990) and Kosaka and Enda (1991). Diet for *M. alternatus* was composed of 8 g of the current and 1‐year‐old needles of *P. densiflora* dried at 90°C for 1 day and milled into powder, 26.8 g of artificial silkworm diet (Silkmate 2M powder, Nosan Corporation, Kanagawa, Japan), 3.2 g of dried yeast (EBIOS, Asahi Group Foods, Ltd., Tokyo, Japan), and 62 ml of distilled water. For *A. luxuriosa* (Maehara et al., 2013), diet consisted of 8 g of leaves of *A. elata* dried at 70°C for 1 day and milled into powder, 26.8 g of Silkmate 2M powder, 3.2 g of dried yeast, and 62 ml of distilled water. For *P. hilaris* (Maehara & Kanzaki, 2016), diet contained 8 g of leaves of *M. bombycis* dried at 70°C for 1 day and milled into powder, 26.8 g of Silkmate 2M powder, 3.2 g of dried yeast, and 62 ml of distilled water. Approximately 20 g of each diet was placed into 50‐ml Erlenmeyer flasks. Flasks were plugged with a silicone‐rubber stopper (Silicosen, Shin‐Etsu Polymer Co., Ltd., Tokyo, Japan) and autoclaved at 121°C for 20 min. A hatched larva of *M. alternatus*, *A. luxuriosa*, or *P. hilaris* was placed into each flask. Larvae were reared at 25°C in the dark for 3–5 months. When mature, larvae were incubated at 10°C in the dark for 9 months. Larvae were subsequently removed from the flasks, rinsed in distilled water, dipped in 70% ethanol for 5 s, and then rinsed again in distilled water. The larvae for use in the first experiment were placed on wet filter paper with distilled water at 25°C in the dark until they pupated. Beetles for the second experiment were reared for one more generation in the same manner.

Mature larvae of *A. fraudatrix* were collected in April and May 2010 from *P. thunbergii* logs in Fukaura, Aomori, Japan and kept at 10°C in the dark in 15‐ml centrifuge tubes with wet filter paper. Most of them pupated at 10°C. Remaining larvae pupated only after incubation at 25°C in the dark in the first experiment. For the second experiment, in summer 2010, some adults of *A. fraudatrix* reared from the larvae were allowed to oviposit on *Larix kaempferi* (Lamb.) Carrière logs that were cut about 1 month prior. After the frass of beetles was found on the logs, larvae were collected and placed into flasks with the artificial diet for *M. alternatus*. Larvae were reared at 25°C in the dark for 6–8 months and, when mature, were incubated at 10°C in the dark for 5–7 months. Larvae were subsequently treated in the same manner as larvae of *M. alternatus*, *A. luxuriosa*, and *P. hilaris* until they pupated.

### 2.2 Nematode cultures

Four species of *Bursaphelenchus* were used in the first experiment: a virulent isolate (T‐4) of *B. xylophilus* isolated from a dead *P. densiflora* tree in Ichinoseki, Iwate, Japan in 1992 by T. Kiyohara (Aikawa et al., 2003); *B. luxuriosae* obtained from *A. luxuriosa* in Gose, Nara, Japan (Kanzaki & Futai, 2003a); *B. conicaudatus* isolated from *P. hilaris* in Kyoto, Kyoto, Japan (Kanzaki et al., 2000); and *B. doui* obtained from *M. subfasciatus* found at the Tama Forest Science Garden, FFPRI, Hachioji, Tokyo, Japan (Kanzaki et al., 2008). In the second experiment, two species of *Bursaphelenchus* were used: *B. xylophilus* (T‐4) and *B. mucronatus* subsp. *kolymensis* (Braasch et al., 2011) obtained from *M. saltuarius* in Kyoto, Kyoto, Japan (Hosoda, 1973).

Nematodes were reared on *Botrytis cinerea* Pers. grown on autoclaved barley grains at 20°C in the dark for 9–16 days in the first experiment and at 25°C in the dark for 15 days in the second experiment, and were isolated aseptically from the culture using the Baermann funnel technique (Hooper, 1986). A nematode inoculum was prepared with 500 nematodes/30 μl suspension.

### 2.3 Loading beetles with nematodes on fungal plates

**FIGURE 1:**
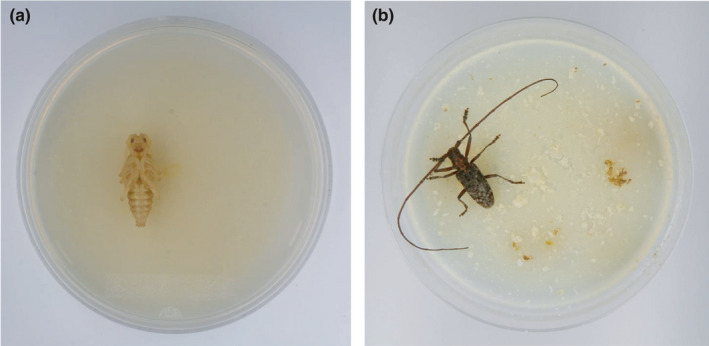
Simple nematode‐loading method to cerambycid beetles. (a) A pupa and (b) an adult of *Monochamus alternatus*

Mycelial disks (4 mm in diam.) of *Nectria viridescens* Booth, cut from fungal colonies growing on potato dextrose agar (Difco), were placed on malt extract agar (Difco) (5% agar) in 9 cm diam. Petri dishes. These dishes were incubated at 25°C in the dark for 20 days. A 30 μl nematode suspension (= 500 mixed‐stage nematodes) was inoculated into each dish and incubated at 20°C in the dark for 11 days and subsequently at 25°C in the dark for 22 days in the first experiment, and at 25°C in the dark for 15–20 days in the second experiment. In both experiments, a final incubation at 10°C in the dark continued until larvae of cerambycid beetles pupated. After the pupation, one pupa was placed onto each dish (Figure 1). Control dishes received no pupae. Dishes were wrapped in Parafilm M^®^ (Bemis Flexible Packaging, Wisconsin, USA) and incubated at 25°C in the dark.

The development of pupae was observed daily. Eight days after adult eclosion, adults of the beetles were removed from the dishes. After removal, each beetle was rinsed with distilled water, ground for 10 s using a blender in 40 ml of distilled water, and placed in a Baermann funnel overnight to extract the nematodes in the body. To determine the number of nematodes that were unable to enter beetle tracheae, rinse water from beetles and agar medium were placed in another Baermann funnel overnight. Harvested nematodes were then counted using a stereomicroscope, and J_III_, J_IV_, PA, and all other developmental stages (propagative juveniles and adults) were recorded for each sample. When nematodes were too abundant to count, the suspension was diluted, and the numbers of nematodes were estimated. In the first experiment, we used 16 combinations of four nematode and four beetle species along with four controls with only nematodes. In the second experiment, eight combinations of two nematode and four beetle species were used along with two controls with only nematodes.

### 2.4 Statistical analyses

All analyses were conducted using JMP^®^ 11 (SAS Institute Inc., Cary, NC, USA). The total numbers of nematodes, J_IV_, and PA represent those carried internally by a beetle, and those on the surface of the beetle and remaining in the agar. Two‐way analysis of variance (ANOVA) was used to analyse differences in the total numbers of nematodes, J_IV_, and J_IV_ + PA; the numbers of J_IV_ and J_IV_ + PA carried by a beetle; and the percentages of total J_IV_ and total J_IV_ + PA to total nematodes among beetle treatments. For ANOVA, the numbers of nematodes were log_10_‐transformed, and the percentages of J_IV_ and J_IV_ + PA were arcsine transformed (Yonezawa et al., 1988).

## 3 RESULTS

### 3.1 Loading beetles with nematodes on fungal plates in the first experiment

Table 1 shows phoretic stage formation of four species of *B. xylophilus* group nematodes in four species of cerambycid beetles, and transfer of the nematodes to the beetles in the first experiment. The mean total nematode numbers in fungal plates with and without beetles were greater than 10,000, although the numbers varied widely among treatments and within each treatment (*p* < .0001 for nematodes, beetles, and nematodes × beetles interaction). Phoretic stages of *B. conicaudatus* and *B. xylophilus* were J_IV_, and the stage of *B. luxuriosae* was PA. *Bursaphelenchus doui* had both J_IV_ and PA as its phoretic stages. More than 500 nematodes (J_IV_ + PA) in the mean transferred to beetles: *B. luxuriosae* to both *A. luxuriosa* and *A. fraudatrix*; *B. conicaudatus* to *P. hilaris*; *B. doui* to *A. luxuriosa*, *P. hilaris*, and *A. fraudatrix*; and *B. xylophilus* to *M. alternatus* (*p* < .0001 for nematodes, beetles, and nematodes × beetles interaction). On the other hand, few numbers of *B. luxuriosae* and *B. conicaudatus* transferred to *M. alternatus*. The total numbers of J_IV_ + PA (*p* < .0001 for nematodes, beetles, and nematodes × beetles interaction) and the percentages of total J_IV_ + PA to total nematodes (*p* < .0001 for nematodes, beetles, and nematodes × beetles interaction) showed similar trends to the numbers of J_IV_ + PA carried by a beetle among the plates with beetles. PA of *B. luxuriosae*, J_IV_ of *B. conicaudatus*, and both PA and J_IV_ of *B. doui* did not appear and all nematodes were propagative juveniles and adults in controls without beetles. In contrast, only a few J_IV_ of *B. xylophilus* appeared in controls. Only in *B. doui*, the percentages of beetles carrying nematodes to total beetles were 100% for all four species of beetles, and those in the other nematodes were not always 100% for all beetles.

**Table 1:** Effects of four cerambycid beetle species in the tribe Lamiini on phoretic stage formation of four species of *Bursaphelenchus xylophilus* group nematodes, and transfer of the nematodes to the beetles in the first experiment

Treatment	No. of observations	Total no. of nematodes	No. of J_IV_ carried by a beetle	No. of PA carried by a beetle	No. of J_IV_ + PA carried by a beetle
*B. luxuriosae* + *A. luxuriosa*	6	23,930 ± 9,150	0 ± 0	2,792 ± 2,363	2,792 ± 2,363
*B. luxuriosae* + *P. hilaris*	7	33,449 ± 21,263	0 ± 0	119 ± 152	119 ± 152
*B. luxuriosae* + *A. fraudatrix*	8	23,333 ± 10,888	0 ± 0	1,721 ± 1,712	1,721 ± 1,712
*B. luxuriosae* + *M. alternatus*	11	72,060 ± 13,960	0 ± 0	0.5 ± 0.5	0.5 ± 0.5
*B. luxuriosae*	10	86,980 ± 21,889	–	–	–
*B. conicaudatus* + *A. luxuriosa*	5	36,405 ± 25,171	23 ± 13	0 ± 0	23 ± 13
*B. conicaudatus* + *P. hilaris*	4	13,773 ± 13,580	513 ± 633	0 ± 0	513 ± 633
*B. conicaudatus* + *A. fraudatrix*	10	39,125 ± 21,266	46 ± 59	0 ± 0	46 ± 59
*B. conicaudatus* + *M. alternatus*	11	55,429 ± 18,223	0.09 ± 0.30	0 ± 0	0.09 ± 0.30
*B. conicaudatus*	10	103,840 ± 74,538	–	–	–
*B. doui* + *A. luxuriosa*	7	34,215 ± 12,166	2,159 ± 2,153	189 ± 281	2,348 ± 2,364
*B. doui* + *P. hilaris*	8	15,737 ± 7,752	2,649 ± 3,296	276 ± 368	2,925 ± 3,654
*B. doui* + *A. fraudatrix*	8	62,774 ± 26,080	3,244 ± 3,774	138 ± 215	3,381 ± 3,932
*B. doui* + *M. alternatus*	11	98,981 ± 11,920	110 ± 85	3 ± 3	112 ± 88
*B. doui*	9	105,156 ± 31,458	–	–	–
*B. xylophilus* + *A. luxuriosa*	5	29,593 ± 22,634	24 ± 35	0 ± 0	24 ± 35
*B. xylophilus* + *P. hilaris*	8	15,847 ± 11,040	51 ± 86	0 ± 0	51 ± 86
*B. xylophilus* + *A. fraudatrix*	9	13,443 ± 9,984	3 ± 5	0 ± 0	3 ± 5
*B. xylophilus* + *M. alternatus*	10	16,808 ± 3,778	1,161 ± 863	0 ± 0	1,161 ± 863
*B. xylophilus*	5	37,224 ± 9,282	–	–	–

Treatment	No. of observations	Total no. of J_IV_	Total no. of PA	Total no. of J_IV_ + PA	% total J_IV_ + PA to total nematodes	% beetles carrying nematodes to total beetles
*B. luxuriosae* + *A. luxuriosa*	6	0 ± 0	3,625 ± 2,246	3,625 ± 2,246	16.1 ± 10.5	100.0
*B. luxuriosae* + *P. hilaris*	7	0 ± 0	738 ± 806	738 ± 806	2.3 ± 1.7	100.0
*B. luxuriosae* + *A. fraudatrix*	8	0 ± 0	3,193 ± 2,109	3,193 ± 2,109	14.6 ± 8.6	100.0
*B. luxuriosae* + *M. alternatus*	11	0 ± 0	2 ± 6	2 ± 6	0.003 ± 0.009	45.5
*B. luxuriosae*	10	0 ± 0	0 ± 0	0 ± 0	0 ± 0	–
*B. conicaudatus* + *A. luxuriosa*	5	543 ± 617	0 ± 0	543 ± 617	3.1 ± 4.5	100.0
*B. conicaudatus* + *P. hilaris*	4	2,363 ± 1,028	0 ± 0	2,363 ± 1,028	29.1 ± 23.0	100.0
*B. conicaudatus* + *A. fraudatrix*	10	151 ± 154	0 ± 0	151 ± 154	0.36 ± 0.35	80.0
*B. conicaudatus* + *M. alternatus*	11	0.09 ± 0.30	0 ± 0	0.09 ± 0.30	0.0002 ± 0.0007	9.1
*B. conicaudatus*	10	0 ± 0	0 ± 0	0 ± 0	0 ± 0	–
*B. doui* + *A. luxuriosa*	7	3,842 ± 2,322	480 ± 258	4,322 ± 2,439	13.0 ± 6.3	100.0
*B. doui* + *P. hilaris*	8	4,750 ± 3,847	647 ± 493	5,397 ± 4,235	30.8 ± 15.1	100.0
*B. doui* + *A. fraudatrix*	8	4,489 ± 4,570	220 ± 292	4,709 ± 4,750	11.1 ± 15.6	100.0
*B. doui* + *M. alternatus*	11	640 ± 436	3 ± 3	643 ± 437	0.67 ± 0.49	100.0
*B. doui*	9	0 ± 0	0 ± 0	0 ± 0	0 ± 0	–
*B. xylophilus* + *A. luxuriosa*	5	108 ± 161	0 ± 0	108 ± 161	2.5 ± 5.3	100.0
*B. xylophilus* + *P. hilaris*	8	408 ± 272	0 ± 0	408 ± 272	4.3 ± 6.0	100.0
*B. xylophilus* + *A. fraudatrix*	9	39 ± 52	0 ± 0	39 ± 52	0.80 ± 1.32	55.6
*B. xylophilus* + *M. alternatus*	10	3,241 ± 1,416	0 ± 0	3,241 ± 1,416	20.1 ± 8.7	100.0
*B. xylophilus*	5	24 ± 43	0 ± 0	24 ± 43	0.07 ± 0.11	–

Note

Values are means ± *SD*. J_IV_ and PA represent the fourth‐stage dispersal juveniles and the phoretic adults, respectively. Underlines indicate the combinations which occur under natural conditions.

### 3.2 Loading beetles with nematodes on fungal plates in the second experiment

Table 2 indicates phoretic stage formation data for *B. m. kolymensis* and *B. xylophilus* in four species of cerambycid beetles, and transfer of the nematodes to the beetles in the second experiment. Means of total nematode numbers in fungal plates with and without beetles were more than 10,000, although the numbers of *B. xylophilus* were higher than those of *B. mucronatus* (*p* = .0027 for nematodes, *p* = .8229 for beetles, and *p* = .0641 for nematodes × beetles interaction). Phoretic stages of *B. m. kolymensis* and *B. xylophilus* were J_IV_. Large numbers (mean > 1,000) of *B. xylophilus* transferred to *M. alternatus*, but such large numbers of *B. m. kolymensis* did not transfer to any beetle species (*p* < .0001 for nematodes, beetles, and nematodes × beetles interaction). The total numbers of J_IV_ (*p* = .002 for nematodes, *p* < .0001 for beetles, and *p* = .0004 for nematodes × beetles interaction) and the percentages of total J_IV_ to total nematodes (*p* = .0156 for nematodes and *p* < .0001 for beetles and nematodes × beetles interaction) indicated similar trends to the numbers of J_IV_ carried by a beetle among the plates with beetles. J_IV_ of *B. m. kolymensis* was induced in higher percentage by *P. hilaris* (4.4%) than by *M. alternatus* (1.79%). J_IV_ of *B. m. kolymensis* did not appear and all nematodes were propagative juveniles and adults in controls without beetles. By contrast, only a few J_IV_ of *B. xylophilus* appeared in controls. The percentages of beetles carrying nematodes to total beetles were 100% for *P. hilaris* in *B. m. kolymensis* and for *P. hilaris* and *M. alternatus* in *B. xylophilus*.

**Table 2:** Effects of four cerambycid beetle species in the tribe Lamiini on phoretic stage formation of *Bursaphelenchus mucronatus kolymensis* and *B. xylophilus*, and transfer of the nematodes to the beetles in the second experiment

Treatment	No. of observations	Total no. of nematodes	No. of J_IV_ carried by a beetle	Total no. of J_IV_	% total J_IV_ to total nematodes	% beetles carrying nematodes to total beetles
*B. m. kolymensis* + *A. luxuriosa*	3	29,726 ± 31,354	2 ± 4	169 ± 212	0.67 ± 1.03	33.3
*B. m. kolymensis* + *P. hilaris*	9	16,753 ± 12,777	113 ± 133	724 ± 671	4.4 ± 2.7	100.0
*B. m. kolymensis* + *A. fraudatrix*	6	22,795 ± 6,179	4 ± 5	204 ± 168	0.95 ± 0.75	66.7
*B. m. kolymensis* + *M. alternatus*	13	17,804 ± 6,842	11 ± 32	322 ± 271	1.79 ± 1.50	69.2
*B. m. kolymensis*	10	11,140 ± 4,652	–	0 ± 0	0 ± 0	–
*B. xylophilus* + *A. luxuriosa*	2	35,629 ± 35,208	13 ± 18	125 ± 141	1.1 ± 1.5	50.0
*B. xylophilus* + *P. hilaris*	10	31,044 ± 18,219	64 ± 78	419 ± 543	2.0 ± 2.7	100.0
*B. xylophilus* + *A. fraudatrix*	6	27,185 ± 12,883	36 ± 48	344 ± 276	1.2 ± 1.0	83.3
*B. xylophilus* + *M. alternatus*	14	22,788 ± 11,133	1,945 ± 1,631	3,559 ± 2,233	19.3 ± 13.1	100.0
*B. xylophilus*	9	38,507 ± 14,455	–	40 ± 37	0.11 ± 0.10	–

Note

Values are means ± *SD*. J_IV_ represents the fourth‐stage dispersal juveniles. An underline indicates the combination which occurs under natural conditions.

### 3.3 Affinity of nematodes for beetles

The affinity of five species of *B. xylophilus* group nematodes for four species of cerambycid beetles was based on phoretic stage formation of the nematodes (= the percentage of total J_IV_ + PA to total nematodes) in both the first and the second experiments (Table 3). The phoretic stages of *B. luxuriosae* and *B. conicaudatus*, PA and J_IV_, respectively, were induced by *A. luxuriosa*, *P. hilaris*, and *A. fraudatrix*; these stages were nearly absent in *M. alternatus*. PA and J_IV_ of *B. doui* appeared in all species of beetles examined although the affinity of the nematode for *M. alternatus* was weak. J_IV_ of *B. m. kolymensis* developed in the presence of every species of beetles used; however, the affinity was moderate or weak. In contrast, J_IV_ of *B. xylophilus* appeared in high percentages, 20.1 ± 8.7 (mean ± *SD*) in the first experiment (Table 1) and 19.3 ± 13.1 in the second experiment (Table 2) in *M. alternatus* although the affinity for the other beetles was moderate or weak.

**Table 3:** Affinity of five species of *Bursaphelenchus xylophilus* group nematodes for four cerambycid beetle species in the tribe Lamiini based on the phoretic stage formation of the nematodes in the first and the second experiments

	*A. luxuriosa*	*P. hilaris*	*A. fraudatrix*	*M. alternatus*
*B. luxuriosae*	+++	++	+++	±
*B. conicaudatus*	++	+++	+	±
*B. doui*	+++	+++	+++	+
*B. mucronatus kolymensis*	+	++	+	++
*B. xylophilus*	++	++	+ or ++	+++

Note

+++ (strong), the percentage of total J_IV_ + PA to total nematodes was more than 10%; ++ (moderate), 1%–10%; + (weak), 0.1%–1%; ± (almost no), less than 0.1%.

## 4 DISCUSSION

To be carried by vector beetles, nematodes need to develop into the phoretic stages, because propagative juveniles and adults cannot transfer to beetles even if they are around pupal chambers of the beetles. Therefore, the affinity between nematodes and beetles can be examined by the induction of the phoretic stages in the presence of the beetles. Our simple nematode‐loading method to beetles (Maehara & Kanzaki, 2016) can be used to examine not only the nematodes' affinity for the vector beetles but also the potential affinity for the nonvectors which do not meet the nematodes in the field. Based on the potential affinity, we discussed vector switching of nematodes during the evolutionary history of the *B. xylophilus* group.

The main reason why the mean total numbers of nematodes in the experimental units with and without four species of beetles varied widely among treatments and within each treatment in the first experiment (Table 1) was that the days when the larvae of each beetle species pupated were varied. The nematode populations could have decreased in some dishes where the larvae pupated late. Some beetles were considered to have fed on agar media together with nematodes and to have killed a number of nematodes in the first experiment. Therefore, there were lower total numbers of nematodes in the units with beetles than in the corresponding control units without beetles (Table 1).

More than 500 nematodes in the mean numbers of *B. luxuriosae*, *B. conicaudatus*, *B. doui*, and *B. xylophilus* transferred to their vector beetles, that is, *A. luxuriosa*, *P. hilaris*, *A. fraudatrix* (Table 1), and *M. alternatus* (Tables 1 and 2). In contrast, small numbers of *B. m. kolymensis* transfer to *M. alternatus* (Table 2), because the natural vectors of *B. m. kolymensis* are not *M. alternatus* but *M. saltuarius* (Jikumaru & Togashi, 1995), *M. nitens* (Bates) (Kanzaki & Akiba, 2014), and *M. rosenmuelleri* (Cederhjelm) = *M. urussovii* (Fischer) (Togashi et al., 2008), although the primary vector beetle for *B. m. mucronatus* is *M. alternatus* (Mamiya & Enda, 1979). Several studies reported that J_IV_ of *B. xylophilus* was induced by its vector beetles, *M. alternatus* (Maehara & Futai, 1996, 2001; Ogura & Nakashima, 2002) and *M. carolinensis* (Necibi & Linit, 1998). J_III_ of *B. xylophilus* molt into J_IV_ in response to long‐chain C16 and C18 fatty acid ethyl esters that are secreted from the body surface of *M. alternatus* specifically during adult eclosion (Zhao et al., 2013, 2014). J_IV_ of *B. conicaudatus* and PA of *B. luxuriosae* are also induced by their vectors, *P. hilaris* and *A. luxuriosa*, respectively (Maehara et al., 2013). Moreover, J_IV_ of *B. xylophilus* is induced not only by its vector *M. alternatus* but also by nonvector *P. hilaris* that inhabits not conifers but broad‐leaved trees, although the numbers and the percentages of J_IV_ are higher in the former species (Maehara & Futai, 2001). In the present study, PA of *B. luxuriosae* and both J_IV_ and PA of *B. doui* were equally induced by nonvectors, that is, *A. fraudatrix*, and both *A. luxuriosa* and *P. hilaris*, respectively (Table 1). In the other combinations of five nematode and four nonvector beetle species, the phoretic stages appeared to some extent with the exception of *B. luxuriosae* and *B. conicaudatus* in *M. alternatus* (Tables 1 and 2). Few PA and J_IV_ were recovered from these two combinations. Chemical signals were not identified for induction of the phoretic stages by vectors and nonvectors, except for *B. xylophilus* J_IV_ induction by *M. alternatus* described above (Zhao et al., 2013, 2014). PA of *B. luxuriosae*, J_IV_ of *B. conicaudatus* and *B. m. kolymensis*, and both PA and J_IV_ of *B. doui* did not appear in controls without beetles, while only a few J_IV_ of *B. xylophilus* appeared in controls (Tables 1 and 2). Maehara et al. (2018) also reported appearance of a few J_IV_ of *B. xylophilus* without beetles. Factors involved in the appearance of J_IV_ are not known.

The evolution of the *B. xylophilus* group nematodes from broad‐leaved tree species to species in conifers is indicated by molecular phylogenetic analyses in Figure 3 of Kanzaki et al. (2012). This evolution required cerambycid beetles to transfer nematodes from broad‐leaved trees to conifers. Our hypothesis was “*B. doui* (or its ancestor) was transferred by *A. fraudatrix*, *A. sejuncta*, and/or *M. subfasciatus* (or their ancestral species) from broad‐leaved trees to conifers, switched vectors from these beetles to *Monochamus* beetles, e.g., *M. saltuarius*, in conifers, and then evolved into the common ancestor of *B. mucronatus* and *B. xylophilus*.” We selected *A. fraudatrix* in the present study because the larvae of this beetle are often found in dead pine trees. PA of *B. luxuriosae* and J_IV_ of *B. conicaudatus* were induced by *A. luxuriosa*, *P. hilaris*, and *A. fraudatrix*, but these stages were almost absent in *M. alternatus* (Table 3). This finding indicates that *B. luxuriosae* and *B. conicaudatus* cannot switch their vectors to *Monochamus* beetles. In contrast, PA and J_IV_ of *B. doui* appeared with all four species of beetles examined, although its affinity for *M. alternatus* was weak (Table 3). Moreover, only in *B. doui*, the percentages of beetles carrying nematodes to total beetles were 100% for all four species of beetles. Therefore, *B. doui* (or its ancestor) could switch vectors from *A. fraudatrix* (or its ancestor) to conifer‐using *Monochamus* beetles, e.g., *M. saltuarius*, after transfer by *A. fraudatrix* from broad‐leaved trees to conifers. This idea receives support by the observation that *M. saltuarius* is an actual vector of *B. doui* (Aikawa et al., 2020). The affinity of *B. m. kolymensis* and *B. xylophilus* for *M. alternatus* was stronger than that of *B. doui* for the beetle species (Table 3). Moreover, J_IV_ of the former two species also developed in *A. luxuriosa*, *P. hilaris*, and *A. fraudatrix*, but their affinity for these beetle species was weaker than that of *B. doui*. This observation may reflect vestigial characters from the evolutionary process. These results support our hypothesis that the common ancestor of *B. m. kolymensis* and *B. xylophilus* evolved from *B. doui* (or its ancestor) that switched vectors to *Monochamus* beetles and completed its life cycle in conifers. In addition, J_IV_ of *B. m. kolymensis* was induced in higher percentage by *P. hilaris* than by *M. alternatus*, although these percentages were not so high because both beetles are nonvectors of this nematode (Table 2).

Toki and Kubota (2010) determined the molecular phylogeny of cerambycid beetles in the tribe Lamiini (25 species and 3 additional subspecies in 12 genera) in Japan based on mitochondrial 16S rRNA and cytochrome oxidase subunit I. Kanzaki et al. (2012) developed the molecular phylogenetic analyses of the *B. xylophilus* group inferred from rRNA gene segments D2‐D3 LSU. In addition, Kanzaki and Futai (2002b, 2003b) reported cospeciation between *B. conicaudatus* and its vector beetle, *P. hilaris*. However, relationships between *B. doui* and its four species of vector beetles, *A. fraudatrix*, *A. sejuncta*, *M. subfasciatus*, and *M. saltuarius*, cannot be explained by cospeciation between the nematode and the vectors. We can understand relationships between *B. doui* and its vectors when we consider vector switching of the nematode species based on the wide‐range of affinity of the nematode for cerambycid beetles.

The four species of beetles used in the present study inhabit East Asia, including Japan (Iwata, 1992; Makihara, 1992; Ohbayashi, 1992). Before the Japanese archipelago was separated from the Eurasian Continent in the Miocene (about 20 million years ago) (Santosh & Senshu, 2011), the evolution of *Bursaphelenchus* nematodes through vector switching could have occurred in this continent. Vector switching of *B. xylophilus* actually occurred from *Monochamus* beetles in North America to *M. alternatus* in Japan, and then to *M. galloprovincialis* (Olivier) in Portugal (Akbulut & Stamps, 2012; Ryss et al., 2011). We conclude that vector switching of nematodes could have potentially happened during the evolutionary history of the *B. xylophilus* group.

## CONFLICT OF INTEREST

None declared.

## AUTHOR CONTRIBUTIONS

**Noritoshi Maehara:** Conceptualization (lead); data curation (lead); formal analysis (lead); funding acquisition (lead); investigation (lead); methodology (lead); project administration (lead); resources (equal); writing‐original draft (lead); writing‐review & editing (equal). **Natsumi Kanzaki:** Conceptualization (supporting); data curation (supporting); funding acquisition (supporting); investigation (supporting); methodology (supporting); project administration (supporting); resources (equal); writing‐review & editing (equal). **Takuya Aikawa:** Funding acquisition (supporting); investigation (supporting); methodology (supporting); project administration (supporting); resources (equal); writing‐review & editing (equal). **Katsunori Nakamura:** Funding acquisition (supporting); investigation (supporting); methodology (supporting); project administration (supporting); resources (equal); writing‐review & editing (equal).

## Data Availability

The data used in this paper are deposited in Dryad (https://doi.org/10.5061/dryad.5qfttdz3g).
